# Neutrophil: A New Player in Metastatic Cancers

**DOI:** 10.3389/fimmu.2020.565165

**Published:** 2020-09-24

**Authors:** Mengyue Wu, Mutian Ma, Zhenya Tan, Hong Zheng, Xia Liu

**Affiliations:** ^1^Department of Pathophysiology, Anhui Medical University, Hefei, China; ^2^Department of Toxicology and Cancer Biology, Markey Cancer Center, College of Medicine, University of Kentucky, Lexington, KY, United States

**Keywords:** metastasis, neutrophils, tumor microenvironments, circulating tumor cells, pre-metastatic niche, immunosuppression

## Abstract

The interaction between cancer cells and immune cells is important for the cancer development. However, much attention has been given to T cells and macrophages. Being the most abundant leukocytes in the blood, the functions of neutrophils in cancer have been underdetermined. They have long been considered an “audience” in the development of cancer. However, emerging evidence indicate that neutrophils are a heterogeneous population with plasticity, and subpopulation of neutrophils (such as low density neutrophils, polymorphonuclear-myeloid-derived suppressor cells) are actively involved in cancer growth and metastasis. Here, we review the current understanding of the role of neutrophils in cancer development, with a specific focus on their pro-metastatic functions. We also discuss the potential and challenges of neutrophils as therapeutic targets. A better understanding the role of neutrophils in cancer will discover new mechanisms of metastasis and develop new immunotherapies by targeting neutrophils.

## Introduction

Cancer is a leading cause of death worldwide, and metastasis accounts for the majority of cancer-related mortality. However, the process of metastasis is still poorly understood yet, which has hampered the development of effective anti-metastatic therapies. It is well-known that metastasis is a multiple step process, which includes local tumor cell invasion, entry into the vasculature (intravasation), survival in the circulation followed by the exit from the circulation (extravasation) and colonization at the distal sites. At each step, cancer cells must escape from immune surveillance and killing. Among these immune cells, focus is often given to T cells and macrophages, whereas the functions of neutrophils in cancer have been largely overlooked until recently and still remain elusive (1).

Neutrophils are the most abundant leukocytes in the blood. They have long been considered play an “audience” role in the development of cancer. However, studies of the past decade have found that neutrophils are a heterogenous population including low density neutrophils (LDNs), normal density neutrophils (NDNs), high density neutrophils (HDN), polymorphonuclear-myeloid-derived suppressor cells (PMN-MDSCs) and tumor-associated neutrophils (TANs) (2). Subpopulation of neutrophils plays an important role in cancer progression, particularly in metastasis (3–5). This review summarizes the latest advances in understanding the role of neutrophils in cancer, with a specific focus on their pro-metastatic role. A better understanding the pro-metastatic role of neutrophils will discover new mechanisms of metastasis and open new avenues for immunotherapies by targeting neutrophils.

## Neutrophil Granules, Heterogeneity, and Plasticity

It is well-known that neutrophils play an important physiological role in the body. When the body is inflamed or stressed, neutrophils are always the first to participate in the response, and its unique function is highly dependent on the granules secreted by the cytoplasm. During the maturation of neutrophils, the granules continue to accumulate in the cells, and finally are released when the neutrophils are activated, especially when they participate in the inflammatory response. Neutrophils contain releasable membrane-bound organelles (secretory vesicles), which secrete three main types of cytoplasmic granules: primary granules (azurophilic granules), secondary granules (specific granules) and tertiary granules (gelatinase granules) (6). These granules can be released by exocytosis or mobilized to the surface of neutrophils by envelope fusion (7). Primary granules are the storage of myeloperoxidase (MPO) and most proteolytic enzymes and bactericidal proteins, and are considered as bactericidal chambers mobilized during phagocytosis. Secondary granules are the most numerous granule and contain complement activators and enzymes, e.g., collagenases. Tertiary granules are particularly important in maintaining the homeostasis of the body. They contain biological molecules involved in the physiological processes of neutrophil superoxide anion production, cell adhesion, and diapedesis/extravasation in the early stage of inflammatory reaction, and are rich in matrix metalloproteinase-9 (MMP-9). MMP-9 is considered to be an important participant in the development of cancer (8). It not only plays a critical role in the enzymatic reabsorption and degradation of extracellular matrix (ECM), but also is involved in many other aspects of tumor biology, including angiogenesis, proliferation, invasion and movement, niche formation of cancer stem cells, and promotion of metastatic growth (9, 10). In addition to MMP9, neutrophils also secrete Cathepsin G and Protease 3, and both of them participate in the inflammatory response regulated by neutrophils (11). Cathepsin G and Protease 3 can interact with the receptor for advanced glycation end products (RAGE) but play different roles. The interaction of neutrophil Cathepsin G with tumor cell RAGE is required for neutrophil cytotoxicity to kill tumor cell (12), while neutrophil proteinase 3 interacts with tumor cell RAGE to promote the bone metastasis of prostate cancer (13).

Peripheral blood neutrophils are a heterogeneous population (14, 15), which is evaluated by several parameters including functions (movement, phagocytosis, and oxidative metabolism), physical properties (density, membrane potential), cell surface proteins and enzyme contents. When neutrophils are stimulated and mobilized, neutrophils may express different proteins on the surface of cells, and then change the cell function (16). The physical properties (density, mass, etc.) of neutrophils may also change accordingly (17). After density gradient centrifugation, neutrophils in peripheral blood were clustered. HDNs and NDNs are usually deposited on top of erythrocytes. Peripheral blood mononuclear cells (PBMCs), platelets, immature and activated polymorphonuclear neutrophils (PMNs), LDNs, and PMN-MDSCs are located at the interface between plasma and Ficoll-paque layer. The reported neutrophil subpopulation which promote the pathological process of tumors (occurrence, invasion, and metastasis, etc.) mainly refer to LDNs including mature subtype of LDNs and low density immature PMN-MDSCs (18, 19).

Neutrophils can display either anti-tumor or pro-tumor activity due to heterogeneity and plasticity. Anti-tumor and pro-tumor neutrophils were named as N1 and N2 by Fridlender et al. (20) in 2009. N1 anti-tumor activity is regulated by multiple mechanisms. For example, neutrophils-mediated cancer cell killing is dependent on hepatocyte growth factor (HGF)/MET-induced nitric oxide release (21). N1 neutrophils also can inhibit metastatic seeding in the lungs by generating hydrogen peroxide (H_2_O_2_) which is mediated by tumor secreted chemokine ligand 2(CCL2) (22). Other mechanisms involved in anti-tumor activity of neutrophils include inducing apoptosis of tumor cells by direct contact or release of the tumor necrosis factor-related apoptosis inducing ligand (TRAIL), antibody-dependent cell-mediated cytotoxicity (ADCC), and activation of T cell function (23). On the contrary, N2 neutrophils promote tumor growth and metastasis, which will be discussed in details in the following sections.

Like macrophages (24), N1 and N2 neutrophils can be shifted to each other, depending on specific tumor-derived factors. Transforming growth factor-β (TGF-β) is the well-studied molecule that polarizes neutrophils to N2 phenotype (20). Both TGF-β blockade and neutrophil depletion can induce N1 phenotype and significantly decrease tumor growth in mouse models. Recently, Interleukin (IL) 35 (IL-35) (25) has also been found to promote the neutrophil polarization to N2 phenotype, IL-35 promotes the production of granulocyte-colony stimulating factor (G-CSF) and IL-6, activates signal transducer and activator of transcription 3 (STAT3) and extracellular signal-regulated kinase (ERK) pathways in neutrophils, and increases the expression of inducible nitric oxide synthase (iNOS) to inhibit T cell activity. In addition, studies have shown that type I interferon (IFN) can transform neutrophils into N1 anti-tumor phenotype (26, 27). In the absence of IFN-β, the expression of neutrophil extracellular trap (NET) in primary lesions and pre-migratory lung is low, while the use of interferon therapy in mouse tumor models polarizes neutrophils to N1 anti-tumor phenotype (27). Cytokine concentration and tumor microenvironment (such as hypoxia) changes may also be important for neutrophil polarization. It has been reported that neutrophils display an activated phenotype with anti-tumor role to stimulate T cell responses in the early stage of tumors (28), but with the development of tumors, it gradually becomes N2 phenotype. In addition, N1 neutrophils have a shorter life span, but are more mature and cytotoxic than N2. Therefore, conversion from N2 to N1 phenotypes of neutrophils might be a new way to reactivate anti-tumor immune response of neutrophils.

## Neutrophils: The Linking Between Inflammation and Cancer

Neutrophils are the first responders to sites of infection and tissue damage. Healthy people can produce more than 10^11^ neutrophils per day in the bone marrow (29). Neutrophils are thought to be a short-lived cell type. However, their lifespan is much longer than initially thought and can survive for more than 5 days in the circulation, and may potentially live even for weeks in tissues (30). They are highly mobile and rapidly recruited to infected or injured tissues by multiple signals including cytokines, chemokines, and pathogen signals. Even after the death of neutrophils, its residual DNA structure will form a unique network structure called NET (31, 32), which captures and kills pathogens in the body. However, several lines of evidence have shown that neutrophil functions extend beyond their roles in innate immune response to kill microorganisms, but also contribute to chronic inflammatory conditions and adaptive immune responses, which have been reviewed in detail elsewhere (33–35). Better understanding how neutrophils are recruited and subsequently react to inflammation could help to understand how inflammation induces cancers.

The difference between neutrophils and other immune cells is that they are released from bone marrow as ultimately differentiated mature cells (36). In the different stages of neutrophil differentiation, a variety of cytoplasmic granules with different functions are continuously synthesized, which is the basis for neutrophils to play their cellular functions. Under normal condition, circulating mature neutrophils account for only 1–2% of all neutrophils in the whole body. Mature neutrophils are retained in bone marrow through interaction between two C–X–C chemokine receptors (CXCR): CXCR4 and CXCR2 (37–39). CXC chemokine ligand (CXCL) 12, which is produced by osteoblasts and other bone marrow stromal cells, binds CXCR4^+^ neutrophils to maintain them in the bone marrow. While the secretion of CXCL1 and CXCL2 by endothelial cells and megakaryocytes encourages neutrophils to release into circulation *via* CXCR2 signal (40). Other adhesion molecules such as integrin subunit alpha 4 (ITGα4), vascular cell adhesion molecule 1 (VCAM-1), and some proteases play an important role in neutrophil retention (41, 42). In addition, IL-23 produced by macrophages and IL-17 produced by lymphocytes, can also regulate the release of neutrophils (43).

The presence of white blood cells within tumors was first observed in the 19th century by Rudolf Virchow. It is now recognized that inflammation plays an important role in the occurrence and development of cancer (44–46). Among white blood cells, neutrophils are the common inflammatory cell type infiltrated in many tumors. Albrengues et al. (47) found that persistent experimental pulmonary inflammation induced by exposure to tobacco smoke or nasal instillation of lipopolysaccharide (LPS) and the accompanying formation of NET can wake up dormant cancer cells to transform them into invasive lung metastasis in mice. Awakening of these dormant cancer cells is associated with NET-mediated remodeling of the extracellular matrix. Antonio et al. (48) also found that injury-induced inflammation increases the formation of tumors in a neutrophil-dependent manner in a HrasG12V-driven melanoma zebrafish model. A recent study further suggested that microparticles (such as miR-23a miR-155) released by neutrophils in inflamed tissue can induce genomic instability to promote cancer development (49). Therefore better understanding the correlation between pro-inflammatory function of neutrophils and cancer development could foster a new anti-inflammatory therapeutic approach to treat cancer.

## Early Metastatic Step-Neutrophils at TME

Tumor microenviroment (TME) is the environment surrounding a tumor, which plays an important role for the tumor initiation, growth, and metastasis (50, 51). Multiple immune cell types, including lymphocytes (T cells and B cells) (52, 53), neutrophils (54), macrophages (55), dendritic cells (DCs) (56), and nature killer (NK) cells (57) have been shown to infiltrate the TME. Despite being the major component of the immune system, the contribution of neutrophils to the tumors has been overshadowed by other immune cells such as lymphocytes and macrophages. However, increasing evidence shows that neutrophils also actively participate in regulating TME and tumor progression, which is related to N2 phenotype of neutrophils. This section is focused to discuss the pro-tumor/metastatic role of neutrophils in TME.

Angiogenesis is the physiological process through which the new blood vessels form. However, it is also a fundamental step in the transition of tumors from benign to malignant state. The role of neutrophils in tumor angiogenesis has been previously reviewed (58, 59), and the mechanisms include releasing a variety of proteases (such as MMP9), directly secreting a multitude of soluble pro-angiogenic factors [such as vascular endothelial growth factor (VEGF)], and through intimate cell-to-cell interactions with endothelial cells. In addition, Bv8 is an important mediator of neutrophil-dependent angiogenesis, and blocking Bv8 function can inhibit angiogenesis and tumor growth (60, 61). Recently, transcriptomic analyses demonstrated that pro-angiogenic genes including *vegfa, itgb1, il1b, mmp14*, and *mmp9* as well as genes encoding other cell adhesion molecules, such as *ilgals1* and *igals3bp*, are upregulated in TANs (62), further confirming a prominent role of neutrophils in promoting tumor angiogenesis.

**Figure 1:**
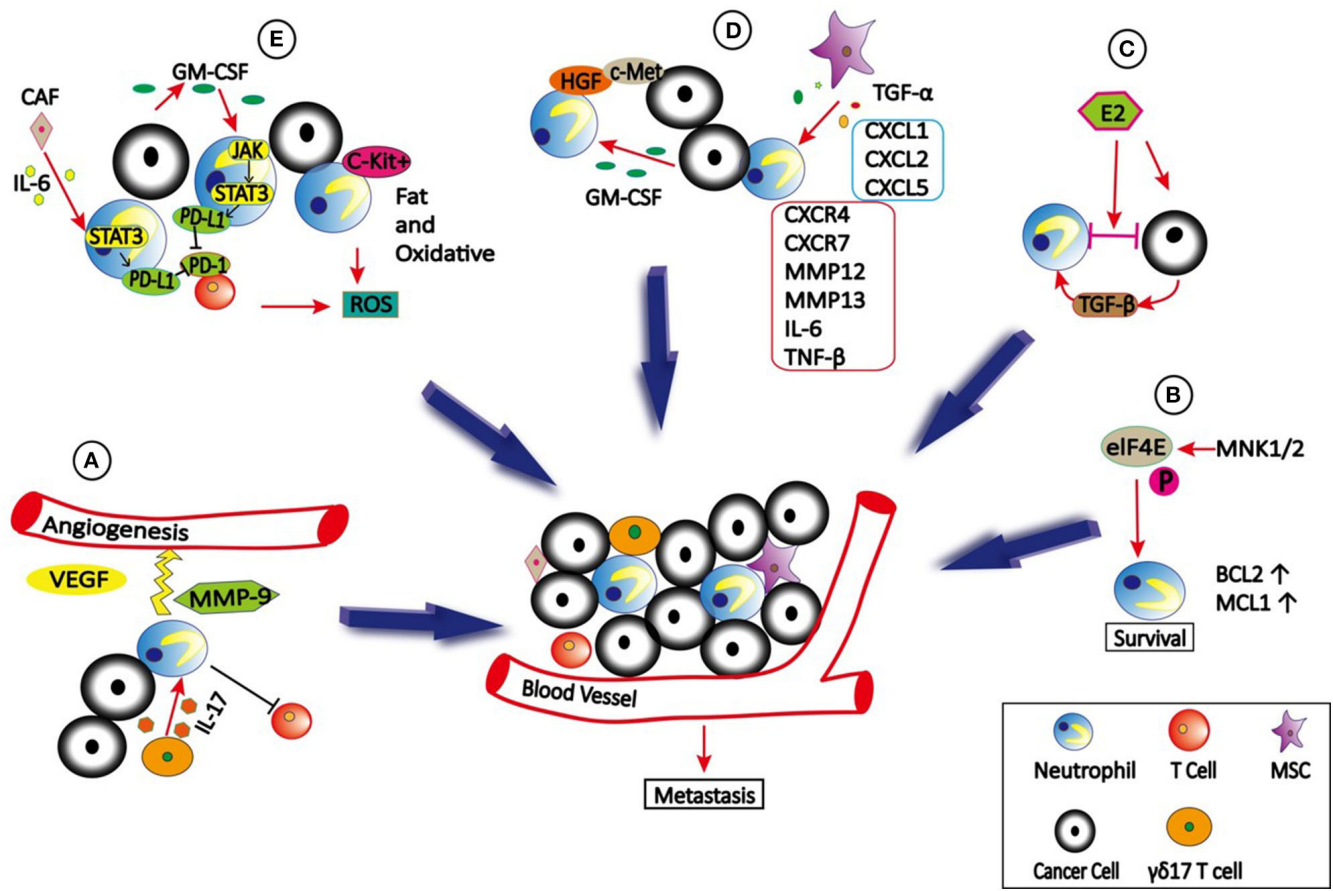
The role of neutrophils in TME. **(A)** γδ17 T cells which secret IL-17 can recruit neutrophils into TME to stimulate the angiogenesis by producing VEGF and MMP9. In addition, γδ17 T cells can produce G-CSF to expand, and polarize neutrophils to promote metastasis by suppressing cytotoxic CD8 T cells immunity. **(B)** eIF4E is phosphorylated by MNK1/2 to promote neutrophils survival and accumulation in TME due to increased expression of BCL2 and MCL1, which then promote metastasis. **(C)** Estradiol (E2) promotes N2 polarization of neutrophils *via* TGF-β1, thus causing cancer cells to be highly metastatic. **(D)** TNFα-activated MSCs promote metastasis *via* production of CXCL1, CXCL2, and CXCL5 to recruit CXCR2^+^ neutrophils, and then neutrophils activate tumor cells to express higher levels of metastasis-related genes including *CXCR4, CXCR7, MMP12, MMP13, IL-6* and *TGF*β. In addition, tumor cells secreted GM-CSF can induce neutrophils to produce HGF, which binds to c-Met on tumor cells to promote metastasis. **(E)** Tumor-derived GM-CSF induces PD-L1 expression on neutrophil *via* JAK-STAT3signaling pathway, and cancer-associated fibroblasts (CAFs) induce PD-L1 expression on neutrophils by IL6—STAT3 signaling pathway, both of which inhibit T-cell immunity. In addition, c-Kit^+^ neutrophils use fatty acid oxidative metabolism to support ROS production to mediate T cell suppression.

Recruitment of neutrophils to the TME is regulated by various cytokines, chemokines and signals. Interleukin 17 (IL-17) is a pro-inflammatory cytokine. Both clinical sample and experimental studies have shown that IL-17 produced by T helper 17 cells can recruit CD15^+^ neutrophils into TME of hepatocellular carcinoma (HCC) by epithelium-derived CXC chemokines (63) (Figure 1). Subsequently, neutrophils stimulate the proangiogenic activity of tumor cells at adjacent invading edges by producing MMP9. Mesenchymal stromal cells (MSCs) can also infiltrate into TME to recruit neutrophils (64). Yu et al. (65) found that Tumor necrosis factor-α (TNF-α)-activated MSCs promote breast cancer metastasis *via* production of CXCL1, CXCL2, and CXCL5 to recruit CD11b^+^Ly6G^+^ CXCR2+ neutrophils, and then neutrophils activate tumor cells to express higher levels of metastasis-related genes including *CXCR4, CXCR7, MMP12, MMP13, IL-6*, and *TGF*β. In HCC, high infiltration of neutrophils in tumor tissues correlates with c-Met-associated worse patient outcomes (66). The infiltrated CD66b^+^ neutrophils are the major source of c-Met ligand hepatocyte growth factor (HGF) in HCCs. Exposure to HCC environments activates neutrophils and the following HGF production, which then enhance the metastasis of malignant cells by HGF/c-met interaction. HCC cell-derived granulocyte-macrophage colony stimulating factor (GM-CSF) is an important determinant in neutrophil HGF production, which in turn enhances the migration and invasion of HCC cells (66). However, c-Met expression on neutrophils induced by tumor-derived TNF-α or other inflammatory stimuli is required for neutrophil chemoattraction and cytotoxicity in response to HGF (21). Consequently, c-Met deletion in neutrophils enhances tumor growth and metastasis. Neutrophils recruited to the TME also actively secrete cytokines and chemokines, which not only enhance their own recruitment but also the recruitment and polarization of other immune cells in the TME, which will be discussed in detail later.

Cell–cell interactions in TME can be enhanced by integrins, which plays a critical role in cancer cell growth and metastasis (67–69). A recent study demonstrated that Estradiol (E2) promotes breast cancer metastasis by enhancing cell–cell interactions between neutrophils and ER^+^ breast cancer cells in TME (70). E2 treatment promoted N2 polarization of neutrophils by inducing expression of the lymphocyte function-associated antigen 1 (LFA-1, CD11a/CD18) integrin *via* elevated transforming growth factor β1 (TGF-β1), leading to cancer cell migration from the primary tumor site to distant organs. Interestingly, these interactions also caused non-metastatic cells to become highly metastatic.

The effect of translation changes on gene expression is much faster than transcriptional alterations (71). Deregulated translation is a hallmark of transformation, uncontrolled cell proliferation, and survival, as well as the metastatic ability of cancer cells. The best-studied translational regulator involved in cancer is the eukaryotic translation initiation factor 4E (eIF4E), which is regulated *via* phosphorylation of serine 209 by the mitogen-activated protein kinase integrating kinases 1/2 (MNK1/2) (72). A recent study showed that translational control by eIF4E in the TME plays a crucial role in metastatic progression. Further investigation indicated that phospho-eIF4E promotes neutrophil survival and accumulation in TME due to increased expression of the anti-apoptotic proteins 2 B-cell lymphoma (BCL2) and myeloid cell leukemia 1(MCL1) (73).

## Circulating Neutrophils, NETs, and Circulating Tumor Cells

Circulating Tumor Cells (CTCs), are tumor cells that have shed into the blood circulation from a primary tumor or metastatic lesions. They are considered “seeds of metastasis.” It is known that CTCs exist as either single cells or CTC clusters which are defined as a group of more than two or three tumor cells. While rare, several recent studies have shown that CTC clusters with enhanced stem cell properties have increased capacity to seed metastases (74–77).

**Figure 2:**
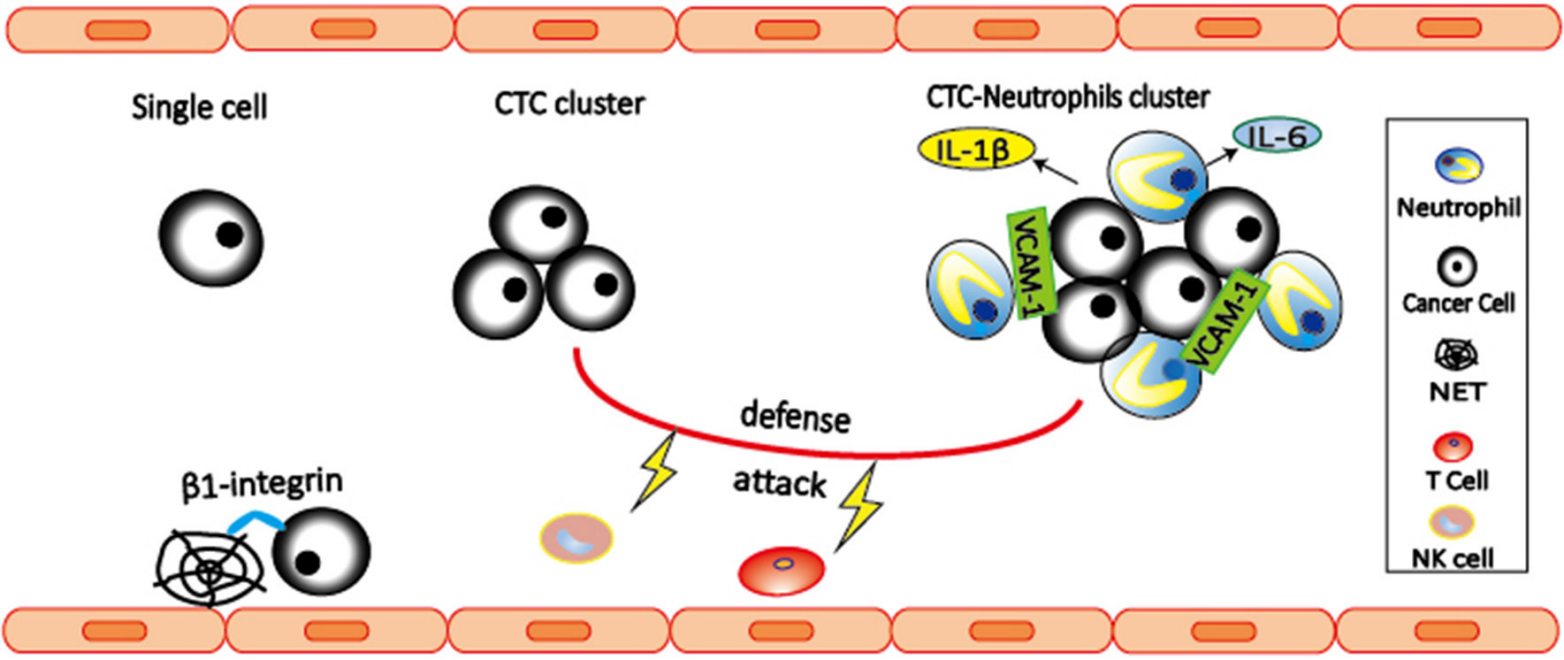
Neutrophils interact with CTCs to promote metastasis. CTC-associated neutrophils express VCAM-1 to mediate their interaction, and produce IL-1β and IL-6 to support CTC proliferation, leading to enhanced metastatic potential of CTC clusters. Both CTC clusters and neutrophil-CTC clusters can prevent CTCs from being killed by NK cells and T cells. In addition, β1-integrin mediated interactions between CTCs and NETs can trap CTCs, thereby promoting adhesion of CTCs to distant organs and metastasis.

Interaction between cancer cells and immune cells is important for the cancer development. However, focus is often given to those interactions that occur within the primary tumor and its microenvironment, and pre-metastatic niche, while the role of immune cells during cancer dissemination in circulation remains largely unknown. CTCs are occasionally found in association with non-malignant cells such as leukocytes (78, 79). By single-cell RNA sequencing of cluster-associated white blood cell (WBC), Szczerba et al. (80) found that the vast majority (85.5–91.7%) of CTC-associated WBCs are CD11b^+^Ly-6G^+^ neutrophils. The association between neutrophils and CTCs drives cell cycle progression to confer proliferative advantage of CTC clusters (80), leading to faster metastasis development and enhanced metastatic potential of CTC clusters. Further RNA sequencing analysis revealed *ARG1, CXCL1, CXCL2, CXCL10, CCL2, CXCR2*, and *VEGFA* are expressed in most CTC-associated neutrophils, indicating that CTC-associated neutrophils are similar to N2 phenotype (Figure 2). CTC-associated neutrophils express VCAM-1 to mediate their interaction with CTCs, and TNF-α, oncostatin M (OSM), IL-1β, and IL-6 to support CTC proliferative advantage. In addition, intercellular adhesion molecule-1 (ICAM-1) was also found to mediate CTCs and neutrophil interaction to promote liver metastasis (81). Based on these two studies, VCAM-1 and ICAM-1 might play the roles at different stages. VCAM-1 mediates interactions between neutrophils and CTCs in the circulation, while ICAM-1 mediates interaction between arrested neutrophils and CTCs to facilitate tumor cells adhesion to the endothelium. Therefore, the link between VCAM-1 and ICAM-1 in metastasis remains to be elucidated in the future studies.

NETs are neutrophil-derived DNA networks released in response to inflammatory signals that trap and kill invading pathogens (82). Several evidences have shown that NETs are involved in trapping CTCs and promote metastasis (83, 84). In 2013, Cools-Lartigue et al. (84) provided the first evidence suggesting that NETs are able to trap CTCs, thereby promoting early adhesion of CTCs to distant organ sites and metastasis. These effects are abrogated by NET inhibition with deoxyribonuclease (DNase) or elastase inhibitor. Later in 2017, Najmeh et al. (83) shed light on the molecular mechanism by which NETs trap CTCs. They found that β1-integrin is an important factor mediating the interactions between CTCs and NETs. Both studies have shown that interactions between CTCs and NETs can be abrogated by DNase, suggesting the potential of NETs as potential therapeutic targets.

CTCs face many survival challenges in the circulation, including immune cell attack, and shear forces. Understanding how CTCs survive will facilitate to develop efficient anti-metastatic treatment. Comparing to single CTCs, CTC clusters have increased expression of NK cell inhibitory ligand HLA and decreased expression of NK cell activating ligand NKG2D, leading to reduced NK cell activation and killing (85). In addition, Zhang et al. (86) found that circulating neutrophils can contribute to CTC survival by suppressing peripheral leukocyte activation to enhance metastasis. Recently, Dr. Millinedo hypothesized (87) that CTCs might interact with neutrophils as a strategy to evade immune detection and attack, which is similar as Leishmania parasites do (88). In this proposed hypothetical model, cancer cells could be transported and protected from immune cell attacks by being surrounded by neutrophils. However, this hypothesis remains to be further investigated.

## Neutrophils in Tumor Cells Extravasation

While CTCs use various strategies to survive in the intravascular environment, their metastatic potential eventually depends on their ability to rapidly extravasate (exit the vessel) into the surrounding tissue. It is well-accepted that extravasation involves arrest in small capillaries and adhesion to the endothelium, rearrangements of the endothelial barrier, and transendothelial migration to the underlying tissues (89). Although extravasation is crucial for metastases, mechanisms underlying this complex process remain to be fully understood.

**Figure 3:**
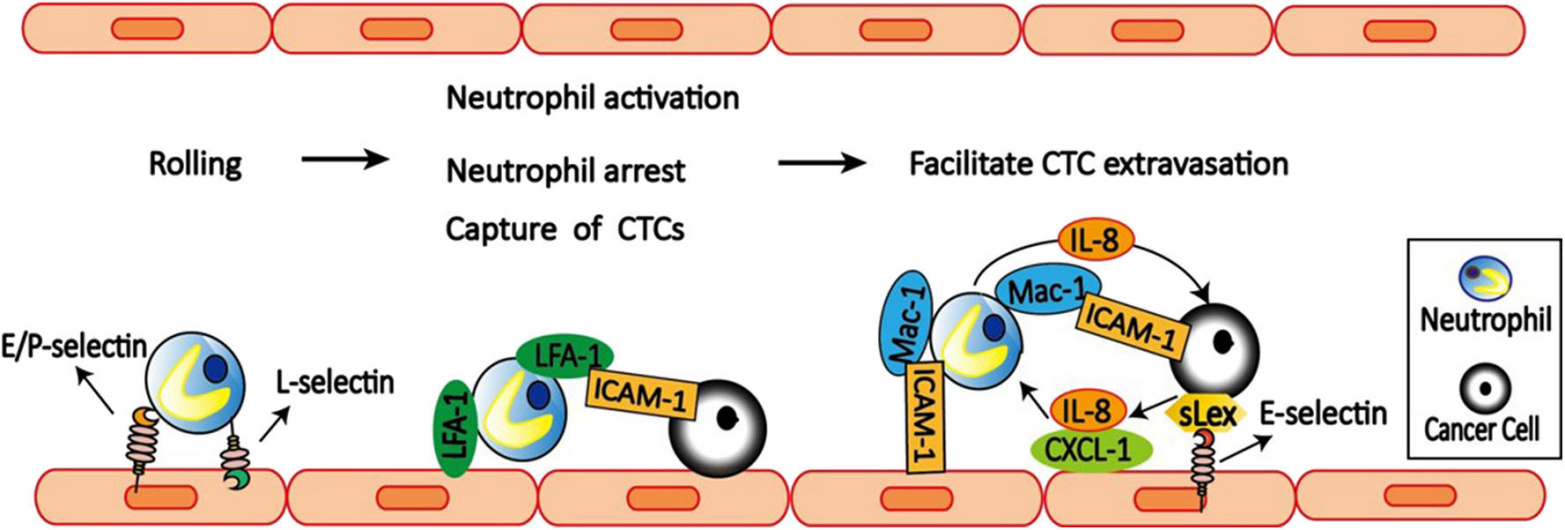
Neutrophils facilitate CTCs extravasation. E**/**P-selectin on the endothelial cell mediates the initial attachment and rolling of neutrophils along the endothelial surface. Then neutrophils capture tumor cells *via* LFA-1 and ICAM-1 interactions. LFA-1 on neutrophils also bind to ICAM-1 on the endothelial cells to arrest and activate neutrophils. After arrest, neutrophils within clusters are highly migratory mediated by neutrophils self-secreted IL-8 and tumor-derived CXCL-1. On the other hand, tumor cell secreted IL-8 increases Mac-1 expression on neutrophils to promote their binding to ICAM-1 on endothelial cells, and also prolong interaction with CTCs *via* Mac-1-mediated interactions with ICAM-1-expressing CTCs. Neutrophils can also facilitate CTCs adhesion to endothelium, which is dependent on selectin ligands containing sialyl-Lewis x (sLex) moieties on the surface of the tumor cells and E-selectin on endothelial cells. Together, neutrophils increase the extravasation potential of nearby entrapped tumor cells through disruption of the endothelial barrier.

Neutrophils participate in the inflammatory response by transendothelial migration, which include three steps: neutrophils rolling, adhesion, and migration (90, 91). Neutrophils rolling is mediated by L-selectin expressed on neutrophils and E-selectin and P-selectin expressed on endothelial cells (Figure 3). More importantly, both neutrophils adhesion and migration require the expression of CD11a/CD18 (LFA-1) and CD11b/CD18 (Mac-1) on neutrophils and the expression of ICAM-1 on endothelial cells (92).

Accumulating evidence suggest that neutrophils can modulate tumor cell attachment to endothelial cells, which is mediated by a mechanism involving direct contact between neutrophils, tumor cells, and the endothelial cell monolayer. Using an *in vivo* model of early metastasis coupled with intravital microscopy, McDonald et al. (93) provided evidence that neutrophils promote metastasis by facilitating CTCs adhesion to liver sinusoidal endothelium, which is dependent on selectin ligands containing Sialyl-Lewis X (sLeX) moieties on the surface of the tumor cells and E-selectin on endothelial cell expression. Consistently, Spicer et al. (81) also found that neutrophils promote cancer cell adhesion within liver sinusoids to promote liver metastasis *via* Mac-1-mediated interactions with ICAM-1-expressing CTCs. Under flow conditions, transmigration of C8161 melanoma cells can be influenced by neutrophils, which is mediated by Mac-1/ICAM-1 adhesive interactions and enhanced by altered IL-8 cytokine production (94). Melanoma cell-secreted IL-8 attracts neutrophils and increases tethering Mac-1 expression on neutrophils to promote anchoring to vascular endothelium (95). However, Mac-1 only affects prolonged neutrophil-melanoma aggregation. While LFA-1 influences the capture phase of neutrophils binding to both melanoma cells and the endothelium (96).

Tumor cells can alter neutrophils function to facilitate their extravasation. For example, tumor-conditioned medium (TCM) downregulates neutrophils cytocidal function, delays neutrophils apoptosis, and upregulates adhesion molecule expression on neutrophils. These neutrophils treated with TCM can attach to tumor cells and facilitate their migration through different endothelial monolayers (97).

To dissect the dynamic roles of inflamed neutrophils during hematogenous dissemination, a multiplexed microfluidic model of the human microvasculature was developed to observe heterotypic tumor cell–neutrophils interactions (98). Surprisingly, neutrophils are not static following aggregation, but exhibit a dynamic migration pattern near tumor cells–neutrophils clusters. The initial clustering of tumor cells and neutrophils is dependent on both physical trapping and adhesive interactions between neutrophils and endothelial ICAM-1. After arrest, neutrophils within clusters are highly migratory, in a confined manner mediated by neutrophils self-secreted IL-8 and tumor-derived CXCL-1. This induces significant neutrophil sequestration with arrested tumor cells, leading to the spatial localization of neutrophil-derived IL-8, and increasing the extravasation potential of nearby entrapped tumor cells through disruption of the endothelial barrier. These findings provide a basis for inhibiting the pro-extravasation effect of neutrophils to reduce metastasis.

In addition, neutrophils can enhance metastasis formation *via* another two distinct mechanisms. First, CD11b^+^Ly6G^+^ neutrophils increase the intraluminal survival time of tumor cells by inhibiting natural killer (NK) cell function (99). Secondly, CD11b^+^ Ly6G^+^ neutrophils expedite extravasation of tumor cells through the secretion of IL1β and MMPs (99). IL1β is a cytokine that is known to be secreted by neutrophils, to activate endothelial cells, and to increase leukocyte extravasation (100). Therefore, these results suggest that neutrophils facilitate tumor transendothelial migration not only through a direct effect on the tumor cells but also through their activation of endothelial cells.

## Neutrophils in Pre-metastatic Niche

After successful extravasation, tumor cells have to survive and outgrow under a novel pre-established and favorable environment in distant organ to form metastasis. This microenvironment is termed pre-metastatic niche by Dr. David Lyden's lab in 2005 (101). Several studies have shown that neutrophils play an important role in this niche to promote metastasis.

**Figure 4:**
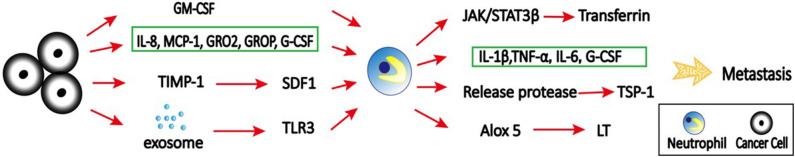
The role of neutrophils in pre-metastatic niche. Primary tumor cells secret various cytokines, chemokines, and signals to recruit neutrophils to distant organs to form pre-metastatic niche. For example, tumor cell secreted-IL-8, MCP-1, GROα, GROβ, and G-CSF can mobilize neutrophils to the pre-metastatic niche, which then facilitate colonization of tumor cells and subsequent metastasis; GM-CSF produced by tumor cells can induce transferrin synthesis in neutrophils through the Jak/Stat5β pathway; TIMP-1 increases SDF1 levels, which in turn promote recruitment of neutrophils to the liver to promote liver metastasis; tumor exosomal RNAs activate TLR3 to induce chemokines that are critical for neutrophil recruitment and lung pre-metastatic niche formation. At pre-metastatic niche, activated neutrophils produce IL-1β, TNF-α, IL-6, and Cox-2, and degranulate azurophilic granules to release elastase and cathepsin G, leading to degradation of the Tsp-1 and enhanced metastasis. In addition, neutrophils support metastatic lung colonization *via* ALOX5-dependent leukotriene (LT) synthesis/release.

Accumulation of neutrophils in the pre-metastatic niche is regulated by various cytokines and chemokines. In colorectal cancer, tissue inhibitor of metalloproteinases-1 (TIMP-1) promotes liver metastasis by triggering the formation of a pre-metastatic niche (Figure 4). This effect is dependent on the increased hepatic stromal cell-derived factor 1 (SDF-1) levels, which in turn promote CXCR4-dependent recruitment of neutrophils to the liver (102). CXCR2-dependent accumulation of Ly6G^+^ neutrophils in the pre-metastatic niche is required for pancreatic ductal adenocarcinoma (PDAC) metastasizing to liver (103). In addition, tumor cell secreted G-CSF can mobilize Ly6G^+^Ly6C^+^ granulocytes to the pre-metastatic niche, which then facilitates colonization of tumor cells and subsequent metastasis (3). The absence of type I IFN can also induce CD11b^+^Ly6G^+^Ly6C^int^ neutrophil-mediated pre-metastatic niche formation due to upregulated CXCR2 expression (104). This finding further support that type I IFN transform neutrophils into N1 anti-tumor phenotype (27). Neutrophil accumulation in lungs supports more efficient tumor cell extravasation and proliferation in the lung due to the enhanced expression of pro-metastatic proteins, such as Bv8, MMP9, S100A8, and S100A9 (104).

Exosomes are a class of extracellular vesicles released by all cells, with a size range of 30–200 nm (105). They play a key role in intercellular communication between cancer cells and their microenvironment through transfer of information *via* their cargo including proteins, DNAs, RNAs, and microRNAs (106–108). Emerging evidence suggests that tumor-derived exosomes can induce the formation of pre-metastatic niche that foster the development of metastatic disease (109, 110). Interestingly, tumor exosomal RNAs activate alveolar epithelial toll-like receptor 3 (TLR3) to induce chemokines (CXCL1, CXCL2, CXCL5, and CXCL12) that are critical for neutrophil recruitment and lung pre-metastatic niche formation (111). Tumor-derived exosomes can also induce N2 polarization of neutrophils to promote gastric cancer cell migration (112) and NETs to establish cancer-associated thrombosis (113).

Inflammation is strongly associated with primary tumor progression, however, little is known about its role in metastatic outgrowth in distant organs. Thrombospondin-1 (Tsp-1) is a secreted ECM protein critical for lung homeostasis and inflammation (114). It has been shown that myeloid cell-derived Tsp-1 contributes to inhibition of metastasis (115). A study from EI Rayes et al. (114) provided mechanistic insights into the contribution of inflammatory neutrophils to metastasis by degradation of Tsp-1. In the study, they demonstrated that inflammation in the lungs leads to the recruitment of bone marrow-derived CD45^+^CD11b^+^Ly6G^+^ neutrophils. These activated neutrophils produce potent inflammatory mediators, including IL-1β, TNF-α, IL-6, and cyclooxygenase-2 (Cox–2), and degranulate azurophilic granules to release elastase and cathepsin G, leading to degradation of the Tsp-1 and enhanced metastasis. Importantly, genetic ablation of these neutrophil proteases can inhibit lung metastasis.

Neutrophils are the major component and driver of metastasis formation within the pre-metastatic niche in mouse breast cancer models (4, 116). Neutrophils infiltrate the metastatic sites before the tumor cells and promote metastasis without effect on the growth of primary tumors. Conditioned medium from pre-metastatic lung neutrophils contains high level of leukotrienes (LTs), the products of the arachidonate 5-lipoxygenase (ALOX5) enzyme, which promote tumor sphere growth *in vitro* and increase the metastatic initiation potential of cancer cells *in vivo* (4). Importantly, ALOX5 inhibitor zileuton can inhibit LT production *in vivo* and reduce spontaneous metastasis, suggesting that targeting LT/ALOX5 is a potential therapeutic strategy to reduce metastasis. However, deletion of ALOX5 in TME promotes lung cancer progression and metastasis by decreasing T cell number (117). These findings suggest that caution should be taken in targeting ALOX5 in lung cancer due to anti-tumorigenic role of ALOX5 in TME through regulation of T cells.

Tumor cells are preferentially colonize and metastasize to specific organs, which is called “organotropic metastasis” (118). A recent study provided the evidence showing that neutrophils are involved in ovarian cancer organotropic metastasis to the omentum by establishing pre-metastatic niche (119). Ovarian tumor–derived inflammatory factors, including IL-8, monocyte chemoattractant protein-1 (MCP-1), growth-regulated oncogene–α and–β (GROα and GROβ), and G-CSF, stimulate neutrophils to mobilize and NET formation in the omentum. NETs, in turn, trap ovarian cancer cells and promote metastasis. Inhibition of NET formation decrease omental colonization, raising the possibility that blockade of NET formation prevents omental metastasis.

The colonized metastatic cells can also promote pro-metastatic function of neutrophils. By analyzing the secretome of neutrophils isolated from tumor-bearing mice, the iron-transporting protein transferrin secreted by Ly6G^+^ neutrophils was identified as the major mitogen for tumor cells (120). GM-CSF produced primarily by tumor cells induces transferrin synthesis in neutrophils through the Janus kinase (Jak)-Stat5β pathway. Depletion of neutrophils, GM-CSF neutralization or inhibition of Jak kinases inhibits lung metastasis and transferrin production in the metastatic microenvironment.

## Neutrophils and Immunosuppression

Immunosuppression is a unique signature of tumor. Several lines of evidence suggest that neutrophils can suppress both innate and adaptive immune response during cancer development and metastasis (121, 122). Tumor cells can reprogram of myeloid differentiation in bone marrow to activate neutrophils. For example, G-CSF can reconstruct the hematopoietic function of bone marrow and promote myeloid differentiation, thus increasing the number of neutrophils with immunosuppressive effect in breast cancer (123). Interestingly, activation of neutrophils in bone marrow of tumor-bearing mice shows two different phases (124). In the mice with early-stage tumor, CD11b^+^Ly6C^lo^Ly6G^+^ neutrophils are lacking of immunosuppressive activity, but have elevated rates of glucose metabolism, increased adenosine triphosphate (ATP) production, and enhanced spontaneous migration. However, in ectopic tumor and late stages of cancer, bone marrow neutrophils demonstrate potent immunosuppressive activity. Since pro-tumor LDNs accumulate continuously with cancer progression (2), it is possible that the functional differences of neutrophils during cancer progression correlates with the transition from HDNs to LDNs, which warrants further investigation.

Neutrophils can also play its immunosuppressive role by degranulation and releasing type 1 arginase (Arg I) to degrade arginine (122). Consistently, Hawila et al. (5) found that CCR5 regulates the immunosuppressive effect of MDSC at tumor sites by enhancing the production of Arg I. Arginine is important for maintain the activity of T-cell through expression T-cell coreceptor CD3ζ, while Arg I can degrade arginine. Therefore, T-cell suppression can be completely restored through pharmacologic Arg I inhibition or with arginine supplementation.

Programmed death ligand 1 (PD-L1, also known as B7-H1 or CD274) is an important co-inhibitory molecule that interacts with programmed death 1 (PD-1) on T cells to block their proliferation, survival and activity (125, 126). In gastric cancer (GC), tumor-derived GM-CSF activates neutrophils and induces PD-L1 expression on neutrophil *via* Jak-Stat3 signaling pathway, therefore inhibiting T-cell immunity and contribute to the GC progression (127). In HCC, PD-L1^+^ neutrophils are accumulated in the peritumoral region of patients, and effectively suppress the proliferation and activation of T cells (128). In addition, cancer-associated fibroblasts (CAFs) can also induce PD-L1 expression on neutrophils to impair T-cell function by IL6 - Stat3 signaling pathway (129) (Figure 1).

Recent study demonstrated that mitochondrial function is correlated with immunosuppressive capacity (130). By characterizing neutrophil mitochondrial function, Rice et al. (131) found that immature, c-Kit^+^ neutrophils can use mitochondrial fatty acid oxidative metabolism to maintain intracellular nicotinamide adenine dinucleotide phosphate (NADPH) levels and support reactive oxygen species (ROS) production to mediate T cell suppression even in the nutrient limited TME. These data suggest that neutrophil mitochondrial metabolism could be an effective target for cancer therapy.

Lymphocyte-derived IL-17 regulates neutrophil expansion *via* induction of G-CSF (132, 133). In breast cancer, IL-17–producing γδ T cells (γδ17 T cells) can produce G-CSF to expand and polarize neutrophils (132). These activated CD11b^+^Ly6G^+^ neutrophils acquire the ability to suppress cytotoxic CD8 T cells, thereby promoting metastasis (134). However, neutrophils can also inhibit pro-tumor γδ17 T cells *via* induction of oxidative stress in the TME (135). Therefore, the interactions between neutrophils and γδ T cells in immunoregulation are complex and warrant further investigation.

Tumor cell-released autophagosomes (TRAP) can suppress T cell activities by inducing IL-10-producing B cells (136). However, the mechanism of TRAP-mediated immune suppression is still largely unknown. A recent study provided a mechanistic insight indicating neutrophils are involved in TRAP-mediated immune suppression (137). TRAPs can be effectively and rapidly phagocytized into neutrophils, and induce neutrophil apoptosis *via* ROS generation and caspase-3 activation. Consequently, the apoptotic neutrophils that have phagocytized TRAPs inhibit the proliferation and activation of T cells in a cell contact- and ROS-dependent manner (137).

Mature neutrophils can acquire a suppressor phenotype after stimulation *via* complement component 3(C3) (138, 139). Complement receptor 3 (CR3; Mac-1; CD11b/CD18) mediates neutrophils recruitment and adhesion by binding to ICAM-1 on endothelial and T cells. C3d plays a central role in the complement system, and enhances antitumor immunity by increasing tumor-infiltrating CD8+ T cells, depleting Tregs, and suppressing PD-1 expression on T cells (140). Ascites from patients with high-grade serous ovarian cancer (HGSOC) can induce the suppressor phenotype in neutrophils from healthy donors (141). However, inhibition of C3 activation abrogates the neutrophils suppressor phenotype (139), supporting the concept of targeting complement to enhance immunotherapy.

## Neutrophils as Therapeutic Targets in Cancer: Challenges and Opportunities

Although the potential to targeting neutrophils in cancer patients has yet to be tested, both preclinical and clinical findings provide a scientific rationale to therapeutic targeting pro-tumor and pro-metastatic neutrophils. Importantly, the cancer field can benefit from already-existing neutrophil-targeting compounds that have been developed for the treatment of inflammatory and autoimmune disease. For example, anti-asthma drug zileuton, an ALOX5 inhibitor, can reduce cancer metastasis in animal models by targeting pro-metastatic neutrophils (4). It would be interesting to assess the correlation between asthma patients treated with zileuton and their cancer risk.

Strategies to targeting neutrophils include the inhibition of their migration by blocking CXCR2 (142, 143). The first clinical trials of CXCR1 and CXCR2 inhibitor, Reparixin, are ongoing in some cancer patients (clinical trial study numbers: NCT02370238, NCT02001974, and NCT01861054). IL-23-IL-17 axis is also involved in neutrophil recruitment (144), which provide another treatment approach. Antagonists targeting IL-23p40 (a subunit of IL-23) and IL-17 have been approved by the US Food and Drug Administration (FDA) for the treatment of psoriasis. Interestingly, IL-17 expression from γδ T cells induces expansion and polarization of neutrophils to promote breast cancer metastasis (134), suggesting γδ T cell/IL-17/neutrophil axis might represent a new strategy to inhibit metastatic disease. As mentioned above, activated neutrophils can induce immunosuppression, so overcoming neutrophil-induced immunosuppression may be one way to synergize current T cell checkpoint inhibitor immunotherapies. Indeed, preclinical studies have shown that combination of anti-PD-1 inhibitor with anti-CXCR2 treatment has synergistic effect to delay tumor growth (145). This concept is also supported by the emerging clinical evidence showing that high neutrophil counts in melanoma patient is associated with poor response to Ipilimumab, a fully monoclonal antibody against human anti-cytotoxic T lymphocyte-associated antigen (CTLA)-4 (146). The inhibitory receptor signal regulatory protein-α (SIRPα) is a myeloid-specific immune checkpoint that engages the “don't eat me” signal CD47 expression. Recent study suggested that anti-human SIRPα antibody, KWAR23, greatly enhances neutrophil and macrophage activity (147).

Although targeting neutrophils appears to be a promising strategy, several challenges particularly related to specificity and safety remain (148). Since their close relationship with other myeloid cells, unselectively targeting neutrophils may have impacts on other immune responses. Therefore, increasing selectivity to target neutrophils are critical for future therapeutic development. Krupa et al. (149) reported one potential approach by conjugating Bruton's tyrosine kinase(Btk) siRNA to an F(ab′)_2_ fragment of an anti-neutrophil monoclonal antibody to selectively inhibit Btk in alveolar neutrophils, highlighting the potential to selectively target neutrophils. In addition, neutrophil-targeting therapeutics must be developed with caution because neutrophils have both anti-tumor and anti-metastatic activities in some experimental setting (20, 22). The possible causes of these discrepancies could be due to neutrophils heterogeneity and plasticity, and the different stages of diseases. Therefore, another strategy is to direct N2 toward N1 neutrophil phenotype conversion. Indeed, TGF-β blockade and TGF-β receptor inhibitors have shown anti-tumor effects in several models (150, 151). Since TGF-β can induce a N2 pro-tumor neutrophil phenotype, stimulation an N1 anti-tumor phenotype by TGF-β blockade and inhibitor could contribute to their effects on tumors. Other challenges include reduced efficacy of other treatment, neutropenia, and susceptibility to infection caused by neutrophils depletion.

## Conclusion

Neutrophils are new players in cancer development and metastasis. However, neutrophils also play anti-tumor/anti-metastatic roles in some cases. The opposite effects of neutrophils in cancers could be due to the heterogeneity and plasticity of neutrophils. In addition, the tumor types and the stages of diseases may also contribute the different functions of neutrophils. Although anti-tumor/metastatic neutrophils do not only have immunosuppressive activity, they are sometimes overlapping with PMN-MDSCs. Because there are no available markers can distinguish these populations. Whether they are different subpopulation of neutrophils, or completely different cell types is still controversial. Identification of specific markers and better characterization of these two types of neutrophils could help to answer these questions.

Neutrophils have a potential role as therapeutic targets (152). New insights into the mechanisms about pro-tumor/pro-metastatic role of neutrophils and neutrophil plasticity will facilitate developing neutrophils-targeting agents and novel therapeutic strategies for patients with advance disease in the near future. However, these agents have to selectively target the pro-tumor/pro-metastatic neutrophil subsets while preserve or boost the anti-tumor/anti-metastatic subsets of neutrophil. Given that neutrophils also play important role in innate immunity, complete suppression of this cell population will put patients at high risk to infections. A promising direction is to identify the regulators and signaling pathways that mediate the tumor-supporting functions of neutrophils and design therapeutics that specifically target these regulators and pathways.

## Author Contributions

MM, MW, and ZT wrote the manuscript. HZ and XL guided the outline and revised manuscript. All authors contributed to the article and approved the submitted version.

## Conflict of Interest

The authors declare that the research was conducted in the absence of any commercial or financial relationships that could be construed as a potential conflict of interest.
